# The Second Wind in McArdle Patients: Fitness Matters

**DOI:** 10.3389/fphys.2021.744632

**Published:** 2021-10-15

**Authors:** Eduardo Salazar-Martínez, Alfredo Santalla, Pedro L. Valenzuela, Gisela Nogales-Gadea, Tomàs Pinós, María Morán, Alejandro Santos-Lozano, Carmen Fiuza-Luces, Alejandro Lucia

**Affiliations:** ^1^Centro de Estudios Universitarios Cardenal Spínola-CEU, Seville, Spain; ^2^Department of Sports and Computing, Pablo de Olavide University, Seville, Spain; ^3^EVOPRED Research Group, Universidad Europea de Canarias, Tenerife, Spain; ^4^Faculty of Sport Sciences, Universidad Europea de Madrid, Madrid, Spain; ^5^Neuromuscular and Neuropediatric Research Group, Institut d’Investigació en Ciències de la Salut Germans Trias i Pujol, Universitat Autònoma de Barcelona, Barcelona, Spain; ^6^Centre for Biomedical Network Research on Rare Diseases (CIBERER), Instituto de Salud Carlos III, Madrid, Spain; ^7^Mitochondrial and Neuromuscular Disorders Unit, Vall d’Hebron Institut de Recerca, Universitat Autònoma de Barcelona, Barcelona, Spain; ^8^Mitochondrial and Neuromuscular Diseases Laboratory, Instituto de Investigación Sanitaria Hospital ‘12 de Octubre’ (‘imas12’), Madrid, Spain; ^9^i+HeALTH, European University Miguel de Cervantes, Valladolid, Spain; ^10^Physical Activity and Health Research Group, Instituto de Investigación Sanitaria Hospital ‘12 de Octubre’ (‘imas12’), Madrid, Spain

**Keywords:** glycogenosis type 5, myophosphorylase deficiency, cardiorespiratory fitness, ventilatory threshold, physical activity second wind in McArdles 2

## Abstract

**Background:** The “second wind” (SW) phenomenon—commonly referring to both an initial period of marked intolerance to dynamic exercise (e.g., brisk walking) that is not followed by perceived improvement and disappearance of previous tachycardia (i.e., the actual “SW”) until 6–10 min has elapsed—is an almost unique feature of McArdle disease that limits adherence to an active lifestyle. In this regard, an increase in the workload eliciting the SW could potentially translate into an improved patients’ exercise tolerance in daily life. We aimed to determine whether aerobic fitness and physical activity (PA) levels are correlated with the minimum workload eliciting the SW in McArdle patients—as well as with the corresponding heart rate value. We also compared the SW variables and aerobic fitness indicators in inactive vs. active patients.

**Methods:** Fifty-four McArdle patients (24 women, mean ± SD age 33 ± 12 years) performed 12-min constant-load and maximum ramp-like cycle-ergometer tests for SW detection and aerobic fitness [peak oxygen uptake (VO_2__peak_) and workload and ventilatory threshold] determination, respectively. They were categorized as physically active/inactive during the prior 6 months (active = reporting ≥150 min/week or ≥75 min/week in moderate or vigorous-intensity aerobic PA, respectively) and were also asked on their self-report of the SW.

**Results:** Both peak and submaximal indicators of aerobic fitness obtained in the ramp tests were significantly correlated with the workload of the SW test, with a particularly strong correlation for the VO_2__peak_ and peak workload attained by the patients (both Pearson’s coefficients > 0.70). Twenty (seven women) and 24 patients (18 women) were categorized as physically active and inactive, respectively. Not only the aerobic fitness level [∼18–19% higher values of VO_2__peak_ (ml⋅kg^–1^⋅min^–1^)] but also the workload of the SW tests was significantly higher in active than in inactive patients. All the inactive patients reported that they experienced the SW during walking/brisk walking in daily life, whereas active patients only reported experiencing this phenomenon during more strenuous activities (very brisk walking/jogging and bicycling).

**Conclusion:** A higher aerobic fitness and an active lifestyle are associated with a higher workload eliciting the so-called SW phenomenon in patients with McArdle disease, which has a positive impact on their exercise tolerance during daily living.

## Introduction

McArdle disease [glycogen storage disease (GSD) type 5] is an autosomal recessive disorder caused by deficiency of the skeletal-muscle isoform of glycogen phosphorylase —“myophosphorylase” — which catalyzes the first step of glycogen metabolism (Santalla et al., 2014). Patients are therefore unable to obtain energy from their muscle glycogen stores. Yet, because the metabolic blockade occurs upstream of the uptake of this substrate into patients’ muscle fibers, the latter can utilize circulating glucose. This disorder is arguably the paradigm of “*exercise intolerance*” (Di Mauro, 2007; Santalla et al., 2014), characterized by the occurrence of early exertional fatigue, together with muscle weakness, myalgia, and muscle contractures associated with exertion, often accompanied by “crises” of rhabdomyolysis (Lucia et al., 2008; Santalla et al., 2014, 2017; Scalco et al., 2020).

Except for a recently described case in phosphoglucomutase type 1 deficiency (GSD type 14; Preisler et al., 2017), a unique feature shown by patients with McArdle disease is the so-called “*second wind*” (SW). Thus, these patients typically experience an initial period of marked intolerance to dynamic exercise (e.g., brisk walking) that is not followed by an improvement (perceived by the patients as an “SW,” with subsequent disappearance of previous tachycardia) after 6–10 min has elapsed (Di Mauro, 2007). Of note, although the SW *per se* (and properly speaking) refers to the aforementioned second phase of improved exercise tolerance, in practical terms (and also herein) the concept of “SW” is commonly used to refer to *both* the initial phase of exercise intolerance and the subsequent SW phase. The SW is easily measurable in the laboratory by simply monitoring the heart rate (HR) response during a constant-load cycle-ergometer test, which can be used as a diagnostic tool of the disease (Di Mauro, 2007). The physiological mechanisms explaining the SW have been previously studied. The first few minutes of exercise act as a warm-up (inducing muscle vasodilation), after which more circulating free fatty acids and especially glucose are available to working muscle fibers; therefore, the upstream blockade in glycogenolysis is partially bypassed, leading to considerable attenuation of early fatigue (Haller and Vissing, 2002). The first phase of the overall SW phenomenon is commonly reported by patients as the need to take a rest when they start a usual exercise task of daily living such as walking (Santalla et al., 2017), and therefore interferes with their normal daily activities and potentially detracts them from leading a more active lifestyle. For instance, the vast majority of all diagnosed Spanish patients report experiencing the SW during daily life (Santalla et al., 2017).

McArdle patients typically show low levels of physical activity (PA; Santalla et al., 2017) and poor aerobic fitness, well below age- and sex-matched normative values (Munguia-Izquierdo and Lucia, 2015) [e.g., mean ± SD values of peak oxygen uptake (VO_2__peak_) of 19.9 ± 6.6 in Spanish patients (Santalla et al., 2017) or median values of 18.5 ml⋅kg^–1^⋅min^–1^ in European patients (Scalco et al., 2020)]. In turn, VO_2__peak_ is significantly higher in physically active patients than in their inactive peers (Santalla et al., 2017) and previous studies have shown the benefits of regular, low-moderate intensity aerobic exercise training (bicycling, brisk walking) to increase the VO_2__peak_ of McArdle patients (Haller et al., 2006; Mate-Munoz et al., 2007; Porcelli et al., 2016). These results are in line with the finding that tailored low-moderate aerobic exercise might be not only safe (Stefanetti et al., 2020) but also useful to improve patients’ physical function in the context of several neurological or neuromuscular disorders, even including degenerative conditions [e.g., amyotrophic lateral sclerosis (Meng et al., 2020) or Becker’s muscular dystrophy (Lanza et al., 2020b)]. Other modalities, such as constant, individually tailored, high-intensity motor training might be effective in patients with degenerative ataxia, including those with severe disease (Lanza et al., 2020a).

However, the influence of aerobic fitness or PA levels on the presentation of the SW remains unknown. This is an important consideration because an increase in the workload eliciting the SW would potentially translate into an improvement in patients’ exercise tolerance during daily life. The purpose of this study was therefore to determine whether aerobic fitness or PA levels are positively correlated with the minimum workload that elicits the SW phenomenon in McArdle patients (as well as with the corresponding HR value obtained) during a cycle-ergometer constant-load test. We also compared SW variables and aerobic fitness indicators in active vs. inactive patients. Our main hypothesis was that aerobic fitness is overall positively associated with the workload eliciting the SW, with the latter being higher in active than in inactive patients.

## Materials and Methods

### Participants

The study protocol was approved by the local ethics committee and all the participants gave their written informed consent. The study was performed from September 2012 to December 2016 in the exercise physiology laboratory of the Universidad Europea de Madrid (UEM). Inclusion criteria were: (i) diagnosis of McArdle disease, as ascertained by the identification of a documented pathogenic mutation in both alleles — whether in homozygosity or heterozygosity — of the gene (*PYGM*) encoding myophosphorylase; (ii) being free of any major cardiorespiratory disease or severe condition contraindicating exercise; (iii) reporting the SW; and (iv) having previous exercise testing experience in our laboratory.

### Exercise Tests

All participants visited the UEM laboratory in the morning after an overnight fast. Before testing, they were categorized as physically active or inactive during the prior 6 months [i.e., active = reporting ≥ 150 min/week in moderate-intensity aerobic PA or ≥75 min/week in vigorous-intensity aerobic PA (Bull et al., 2020)]. Patients were also asked on their self-report of the SW phenomenon (i.e., what type of PA or exercise elicits this phenomenon).

All the tests were performed using the same cycle-ergometer (Ergoselect 220 KL; Ergoline GmbH, Bitz, Germany) and metabolic cart (Vmax 29C; Sensormedics Corp., Yorba Linda, CA, United States), with the latter used to record ECG-determined HR values and expired gas data. First, and based on previous data from each participant, the patients pedaled for ∼3–4 min, and we adjusted the workload (watts) with gentle increases starting from 0 watts (i.e., 5 watts/30 s) such as to identify the minimum workload eliciting an HR value of ≥65% of the age-predicted maximum HR (220 min age, in years). Once this “target” workload was identified (usually in ≤3 min), the participants immediately stopped pedaling and rested for 1 h. Thereafter, they performed a “diagnostic” 12-min constant-load test for SW identification at the identified target workload (Vissing and Haller, 2003). Participants then rested for ∼45 min before they performed a maximum test until exhaustion as detailed elsewhere (Mate-Munoz et al., 2007). The maximum test was preceded by a 5-min free-wheel pedaling warm-up, after which the workload was increased following a ramp protocol (10 watts/min) until volitional exhaustion (Mate-Munoz et al., 2007). The VO_2__peak_ was determined as the highest VO_2_ value (20-s average) recorded during the tests, whereas the workload eliciting the VT was visually identified by two independent investigators (or by a third one in case of disagreement) as the workload eliciting an increase in the 20-s average value of the ventilatory equivalent for oxygen (VE/VO_2_), with no concomitant increase in the ventilatory equivalent for carbon dioxide (VE/VCO_2_; Mate-Munoz et al., 2007).

### Statistics Analysis

Data are expressed as mean ± standard deviation. The distribution of data was studied with the Kolmogorov–Smirnov test. Pearson correlation analyses were used to assess the relationship between aerobic fitness indicators and variables related to the SW phenomenon (i.e., workload of the diagnostic SW test and highest HR value recorded during this test). We also compared SW variables in inactive vs. active patients with the non-parametric Mann Whitney *U* test. The magnitude of the differences was assessed through the computation of effect sizes (Hedge’s g). The level of significance was set at 0.05 and data analysis was performed using the SPSS statistical software package (PASW Statistic 25).

## Results

Fifty-four patients with McArdle disease that met all the inclusion criteria (30 men and 24 women, mean ± SD age 33 ± 12 years [range 11–59], body mass index [BMI] 24.8 ± 4.8 kg⋅m^–2^) agreed to participate in this study. The *PYGM* genotype data are shown in Supplementary File 1.

**FIGURE 1:**
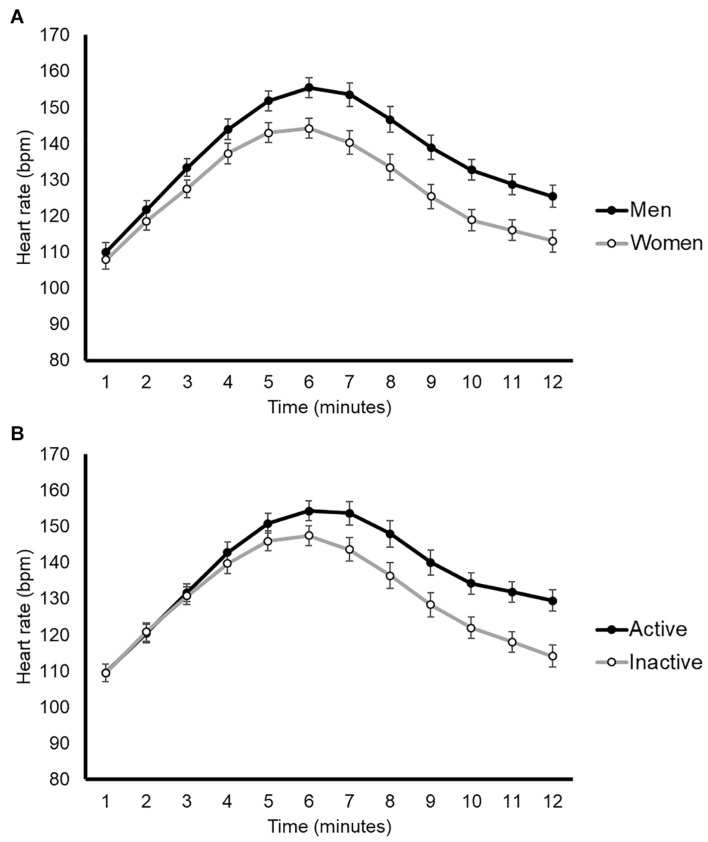
Graphical representation of the heart rate response during second wind tests by sex **(A)** or physical activity levels **(B)**. Data are mean ± SEM (for each 1-min period), with no statistical comparisons done.

**FIGURE 2:**
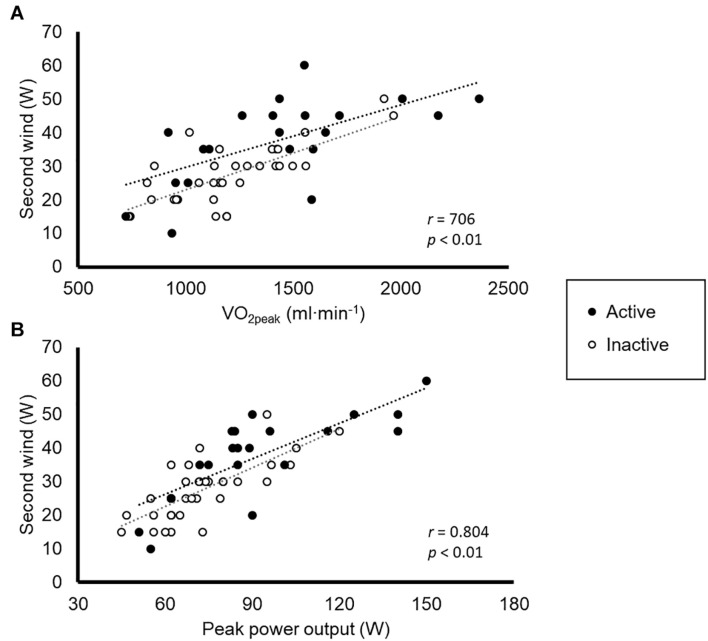
Correlation between peak oxygen uptake (VO_2peak_, **A**) and peak workload **(B)** attained by the patients during the ramp-like tests and the minimum workload eliciting the second wind phenomenon.

The mean physiological responses to the SW and ramp-like tests are shown in Table 1. The HR response in the SW tests is graphically shown in Figure 1. Both peak and submaximal indicators of aerobic fitness obtained in the ramp tests were significantly correlated with the workload of the SW test (Table 2), with a particularly strong correlation found for the VO_2_peak (Figure 2A), and peak workload (Figure 2B) attained by the patients (both Pearson’s coefficients >0.70).

**TABLE 1:** Physiological responses to the second wind and ramp tests in all participants (*n* = 54).

Variable	12-min constant workload test for second wind detection*	Ramp test until exhaustion	
		Ventilatory threshold	Peak values
VO_2_ (ml⋅min^–1^)	864 ± 256	793 ± 229	1,316 ± 356
VO_2_ (ml⋅kg⋅min^–1^)	12.5 ± 3.9	11.2 ± 3.0	18.8 ± 5.0
Power output (watts)	31 ± 11	37 ± 15	82 ± 23
HR (bpm)	147 ± 21	116 ± 15	161 ± 21
V_E_ (l⋅min^–1^)	25.7 ± 10.9	20.2 ± 5.9	41.8 ± 15.5
RER	0.84 ± 0.07	0.82 ± 0.09	0.91 ± 0.12
% of age-predicted HRmax	79 ± 11	73 ± 9	86 ± 10

*Abbreviations: HR, heart rate; HRmax, maximum heart rate (age predicted = 220 min age in years); RER, respiratory exchange ratio; V_*E*_, pulmonary ventilation; and VO_2_, oxygen uptake.*

Symbol: *Average values for all variables correspond to the highest 10-s value of HR obtained during the constant-load test for second wind detection (which on average was reached at 6 min 35 ± 94 s).

**TABLE 2:** Correlation between aerobic fitness (obtained in ramp-like tests) and second wind (SW) variables.

Aerobic fitness indicators	Power output (watts) in SW tests	Highest HR^†^ (bpm) value attained in SW tests
VO_2_peak (ml⋅min^–1^)	0.706**	0.173
VO_2__peak_ (ml⋅kg⋅min^–1^)	0.449**	0.306*
Peak power output (watts)	0.804**	0.081
VO_2_ @VT (ml min^–1^)	0.613**	0.137
VO_2_ @VT (ml⋅kg^–1^ min^–1^)	0.356**	0.279*
Power output @VT (watts)	0.613**	0.042

*Data are Pearson’s correlation coefficients. Abbreviations: HR, heart rate; VO_2__*peak*_, peak oxygen uptake; and VT, ventilatory threshold.*

Symbols: *p < 0.05; **p < 0.01; ^†^10-s averages.

Twenty (7 women) and 24 patients (18 women) were categorized as physically active and inactive, respectively. Not only the aerobic fitness level — notably, ∼18–19% higher values of VO_2__peak_ in ml⋅kg^–1^⋅min^–1^ — but also the workload of the SW tests was significantly higher in active than in inactive patients (Table 3). These results remained overall unchanged in separate analyses for female (Supplementary File 2) or male patients (Supplementary File 3), respectively. On the other hand, all the inactive patients reported that they experienced the SW during walking or brisk walking in daily life whereas active patients only reported experiencing this phenomenon during more strenuous activities, such as very brisk walking/jogging or bicycling.

**TABLE 3:** Physiological responses to the second wind and ramp tests in physically active (*n* = 20; 7 women) vs. inactive participants (*n* = 34; 18 women).

Variable	12-min constant load test for second wind detection*				Ramp test until exhaustion							
					Ventilatory threshold**				Peak values			
	Active	Inactive	*p*-value between groups	ES (Hedge’s g)	Active	Inactive	*p*-value between groups	ES (Hedge’s g)	Active	Inactive	*p*-value between groups	ES (Hedge’s g)
% VO_2__peak_	71 ± 17	66 ± 12	0.425	0.35	65.1 ± 14.8	60.7 ± 11.1	0.226	0.34	–	–		–
VO_2_ (ml⋅kg^–1^⋅min^–1^)	14.6 ± 3.6	11.5 ± 3.5	**0.003**	0.86	13.5 ± 3.2	10.2 ± 2.1	**0.008**	1.27	21.1 ± 4.7	17.4 ± 4.6	**<0.001**	0.78
Power output (watts)	38 ± 12	28 ± 10	**0.002**	0.91	47.8 ± 18.6	31.0 ± 10.2	**<0.001**	1.19	93 ± 27	75 ± 19	**0.008**	0.79
HR (bpm)	155 ± 16	144 ± 2	0.076	1.1	125 ± 12	111 ± 15	**0.001**	0.98	168 ± 20	156 ± 20	**0.044**	0.59
V_E_ (l⋅min^–1^)	28.1 ± 9.3	24.8 ± 7.9	0.145	0.38	21.7 ± 6.8	19.5 ± 4.1	0.245	0.41	45.4 ± 20.8	40.1 ± 12.3	0.546	0.32
RER	0.85 ± 0.05	0.84 ± 0.08	0.490	0.13	0.81 ± 0.08	0.81 ± 0.10	0.808	0.01	0.91 ± 0.1	0.91 ± 0.13	0.932	0.00
% age-predicted HRmax	82 ± 11	78 ± 11	0.217	0.35	75 ± 11	72 ± 9	0.344	0.30	89 ± 12	85 ± 9	0.168	0.38

*Data are presented as Mean ± SD and Hedge’s g effect size (ES). Abbreviations: HRmax, maximum heart rate (age predicted = 220 min age in years); RER, respiratory exchange ratio; VE, pulmonary ventilation; VO_2_, oxygen uptake; VO_2__*peak*_, peak oxygen uptake.*

Symbols: *average values for all variables correspond to the highest 10-s value of HR obtained during the constant-load test for second wind detection (which on average was reached at 6 min 35 s ± 94 s); **detectable in all patients.

*Significant p-values are in bold.*

## Discussion

The main finding of this study was the association between aerobic fitness indicators (VO_2__peak_ and VT) and the workload eliciting the SW in patients with McArdle disease, as well as the fact that the latter corresponded to considerably higher power output levels (by 35%) in active than in inactive patients. This had a translation into patients’ daily life, as in fact corroborated by their own self-reports — that is, active patients did not experience the SW during non-strenuous activities in daily living. Therefore, the present findings would support the importance of increasing PA levels and improving aerobic fitness in this patient population. On the other hand, the main limitation of our study is the cross-sectional design we used, whereas a major strength is the large sample of patients we assessed and the novelty of the question addressed.

Recent research from our group has shown that McArdle patients who are physically active are 14-fold more likely to report an improvement after a 4-year period in the clinical course of the disease compared with their inactive peers, with higher VO_2__peak_ values reported in physically active patients (20.7 ± 6.0 ml⋅kg^–1^⋅min^–1^) than in their inactive referents (16.8 ± 5.3 ml⋅kg^–1^⋅min^–1^; Santalla et al., 2017). In this context, our data also provide additional rationale supporting the need to increase the aerobic fitness as well as the levels of aerobic PA in patients with McArdle disease because a previously unreported benefit associated with a higher aerobic fitness level and an active lifestyle is an increase in the workload eliciting the SW. This is an important consideration when keeping in mind that aerobic (or “cardiorespiratory”) fitness (commonly assessed as VO_2__peak_) is a strong prognostic factor of morbidity and mortality from all causes and, particularly, from cardiometabolic disease (Lavie et al., 2019). Recent data from the European registry of patients with McArdle disease indicate a rather unhealthy cardiometabolic profile for these patients, with two-thirds of them showing high BMI values and ∼12% having cardiovascular disease (8% with coronary artery disease) despite the relatively young age of the cohort (median age 46 years), likely reflecting a sedentary lifestyle related to the poor exercise tolerance and muscle pain on exertion (Scalco et al., 2020).

One of the hallmarks of McArdle disease (which was also corroborated here) is the very poor aerobic fitness of affected patients (Munguia-Izquierdo and Lucia, 2015; Santalla et al., 2017; Scalco et al., 2020). Traditionally, a major reason for this phenomenon has been attributed to an impairment of the patient’s muscle oxidative capacity because the ability to produce pyruvate — a molecule that plays an anaplerotic role in the Krebs cycle — is severely reduced (Lucia et al., 2008). The impairment in the rate of oxidative phosphorylation is reflected on phosphorus magnetic resonance spectroscopy (31P-MRS) by remarkably greater phosphocreatine consumption and lower ATP concentrations compared to healthy controls after submaximal isometric calf contractions (Zange et al., 2003). The resultant marked decrease in skeletal muscle phosphorylation potential ([ATP]/[ADP][phosphate]) leads to the accumulation of phosphate, and probably also ADP, in patients’ muscles, thereby inhibiting myofibrillar ATPase, calcium pump, and sodium-potassium pump reactions and leading to premature muscle fatigue and contractures (Lewis and Haller, 1986). Yet, recent findings from our group in the mouse model of the disease would indicate that myophosphorylase deficiency causes no major alterations in muscle oxidative phosphorylation capacity or autophagy/ubiquitination pathways, thereby suggesting that the patients’ muscle tissue is likely to adapt overall favorably to regular exercise (Fiuza-Luces et al., 2016). In fact, Haller et al. (2006) have shown improvements after training in patients’ muscle oxidative capacity (i.e., increase in the activity of the mitochondrial enzymes citrate synthase and hydroxyacyl coenzyme A dehydrogenase).

Besides the prognostic value of the VT in diseased people in general (Meyer et al., 2005), the occurrence of the VT (sometimes known as “anaerobic threshold”) in McAdle patients is an interesting finding. Indeed, despite not showing lactic acidosis during exercise due to the blockade of glycogenolysis — blood lactate values only average ∼2 mmol⋅l^–1^ at the end of a ramp-like test until exhaustion like the one used here (Mate-Munoz et al., 2007) — these patients show a threshold-like ventilatory response similar to that of healthy people, as shown in a classic study (Whipp, 1983) and corroborated by us both in previous research (Mate-Munoz et al., 2007) and in the present study. Thus, the hyperventilation that accompanies high-intensity exercise may be the result of some mechanism other than acidosis or lung CO_2_ flux (Hagberg et al., 1990), with these patients maybe exhibiting a mechanism for the ventilatory drive which compensates for the lack of blood acidosis. One of such mechanisms might be adenosine released from muscles (Rubio et al., 2008), with extracellular adenosine able to stimulate ventilation not only through activation of carotid chemoreceptors but also through an effect mediated by sensory lung receptors innervated by unmyelinated vagal C fibers (Burki et al., 2005).

In summary, a higher aerobic fitness and an active lifestyle are associated with a higher workload eliciting the so-called SW phenomenon in patients with McArdle disease, which in turn has a beneficial impact on their daily living — that is, improved exercise tolerance. Interventional research is needed to determine which is the best type of exercise/PA intervention to elicit the largest possible improvements in the minimum workload eliciting the SW.

## Data Availability Statement

The original contributions presented in the study are included in the article/Supplementary Material, further inquiries can be directed to the corresponding author/s.

## Ethics Statement

The studies involving human participants were reviewed and approved by Hospital 12 de Octubre. The patients/participants provided their written informed consent to participate in this study.

## Author Contributions

ES-M, AS, CF-L, and AL: data collection (exercise evaluations). AS and AL: conception. GN-G and TP: data collection (molecular genetics for diagnosis). PV, AS-L, and MM: data analysis. MM: supplying of important material. ES-M, AS, and AL: drafting of the manuscript. All authors provided important feedback for the manuscript edition.

## Conflict of Interest

The authors declare that the research was conducted in the absence of any commercial or financial relationships that could be construed as a potential conflict of interest.

## Publisher’s Note

All claims expressed in this article are solely those of the authors and do not necessarily represent those of their affiliated organizations, or those of the publisher, the editors and the reviewers. Any product that may be evaluated in this article, or claim that may be made by its manufacturer, is not guaranteed or endorsed by the publisher.
