# Supplementary material for: The Second Wind in McArdle Patients: Fitness Matters
Source: Front Physiol. 2021 Oct 15;12:744632. doi: 10.3389/fphys.2021.744632 (PMC8555491; doi:10.3389/fphys.2021.744632)
Supplement: Supplementary file 1 [file Data_Sheet_1.docx]

**Supplementary file 1**. Pathogenic *PYGM* genotype indicative of McArdle disease identified in all the participants (*N* = 54)

| **Type of mutation** | ***N*** |
| --- | --- |
| p.R50X (c.148C > T) / p.R50X (c.148C > T) | 19 |
| p.R50X (c.148C > T) / p.W798R (c.2392 T > C) | 6 |
| p.G205S (c.613G > A) / p.G205S (c.613G > A) | 5 |
| p.R50X (c.148C > T) / p.G205S (c.613G > A) | 2 |
| p.R50X (c.148C > T) / p.K754fsX49 (c.2262delA) | 2 |
| p.C784X (c.2352C > A) / p.R94W (c.280C > T) | 1 |
| p.R50X (c.148C > T) / p.R94W (c.280C > T) | 1 |
| p.R50X (c.148C > T) / p.R602W (c.1804C > T) | 1 |
| p.R50X (c.148C > T) / p.A660D (c.1979C > A) | 1 |
| p.R50X (c.148C > T) / p.E383K (c.1147G > A) | 1 |
| p.G205S (c.613G > A) / c.1768 + 1G > A | 2 |
| p.R50X (c.148C > T) / c.1768 + 1G > A | 1 |
| p.R50X (c.148C > T) / p.A365V (c.1094C > T) | 1 |
| p.R50X (c.148C > T) / p.A55GfsX21 (c.163_167delGCTCT) | 1 |
| p.R50X (c.148C > T) / p.A704V (c.2111C > T) | 1 |
| p.R50X (c.148C > T) / p.D534fsX5 (c.1601delA) | 1 |
| p.R50X (c.148C > T) / p.L5VfsX22 (c.13_14delCT) | 2 |
| p.R50X (c.148C > T) / p.R194W (c.580C > T) | 2 |
| p.R50X (c.148C > T) / p.R715W (c.2143C > T) | 1 |
| p.R50X (c.148C > T) / p.T488 N (c.1463C > A) + p.K215 K (c.645G > A) | 2 |
| p.R576X (c.1726C > T) / p.G136AfsX159 (c.407G > A) | 1 |

**Supplementary file 2.** Physiological responses to the second wind and ramp tests in physically active female patients (n=7) *vs* their inactive referents (n=17).

| **Variable** | **12-minute constant load test for second wind detection*** | | | | **Ramp test until exhaustion** | | | | | | | |
| --- | --- | --- | --- | --- | --- | --- | --- | --- | --- | --- | --- | --- |
|  |  |  |  |  | **Ventilatory threshold** | | | | **Peak values** | | | |
|  | **Active** | **Inactive** | **p-value between groups** | **ES (Hedge’s g)** | **Active** | **Inactive** | **p-value between groups** | **ES (Hedge’s g)** | **Active** | **Inactive** | **p-value between groups** | **ES (Hedge’s g)** |
| % of VO_2_ peak  (ml∙min^-1^) | 64.4±19.2 | 66.9±11.7 | 0.707 | 0.17 | 69.2±13.5 | 64.4±13.5 | 0.468 | 0.34 | - | - | - | - |
| VO_2_  (ml∙kg∙min^-1^) | 12.2±2.2 | 10.9±3.4 | 0.420 | 0.40 | 13.5±3.4 | 10.1±2.2 | 0.012 | 1.27 | 20.1±5.8 | 16.4±4.8 | 0.147 | 0.70 |
| Power output (watts) | 31.6±12.5 | 24.1±5.6 | 0.056 | 0.89 | 47.5±25.1 | 27.1±8.4 | 0.007 | 1.31 | 86.3±30.9 | 65±9.4 | 0.017 | 1.14 |
| HR  (bpm) | 149.6±15.5 | 144.1±20.1 | 0.547 | 0.28 | 125.8±15.5 | 113.1±15.5 | 0.102 | 0.79 | 170.5±13.7 | 152.5±16.7 | 0.029 | 1.09 |
| V_E_  (l∙min^-1^) | 26.1±14.1 | 23.2±5.8 | 0.482 | 0.31 | 21±7.9 | 17.9±3.6 | 0.224 | 0.58 | 44.1±24.1 | 34.5±6.9 | 0.155 | 0.66 |
| RER | 0.84±0.08 | 0.83±0.10 | 0.871 | 0.10 | 0.82±0.08 | 0.8±0.12 | 0.718 | 0.17 | 0.93±0.07 | 0.87±0.13 | 0.314 | 0.49 |
| % age-predicted HRmax | 77.8±11.5 | 79.1±9.2 | 0.782 | 0.12 | 73.7±6.7 | 74.4±8.2 | 0.860 | 0.09 | 88.3±7.6 | 83.9±8.5 | 0.279 | 0.51 |

Abbreviations: HRmax, maximum heart rate (age predicted = 220 minus age in years); RER, respiratory exchange ratio; V_E_, pulmonary ventilation; VO_2_, oxygen uptake. VO_2peak_, oxygen uptake. Symbol: * Average values for all variables correspond to the highest 10-second value of HR obtained during the constant-load test for second wind detection (which on average was reached at 6 min 35 s ± 94 s).

**Supplementary file 3.** Physiological responses to the second wind and ramp tests in physically active male patients (n=13) *vs* their inactive referents (n=17).

| **Variable** | **12-minute constant load test for second wind detection*** | | | | **Ramp test until exhaustion** | | | | | | | |
| --- | --- | --- | --- | --- | --- | --- | --- | --- | --- | --- | --- | --- |
|  |  |  |  |  | **Ventilatory threshold** | | | | **Peak values** | | | |
|  | **Active** | **Inactive** | **p-value between groups** | **ES (Hedge’s g)** | **Active** | **Inactive** | **p-value between groups** | **ES (Hedge’s g)** | **Active** | **Inactive** | **p-value between groups** | **ES (Hedge’s g)** |
| % of VO_2_ peak  (ml∙min^-1^) | 73.5±15.9 | 65.1±13.4 | 0.125 | 0.56 | 63.2±15.4 | 56.9±6.4 | 0.141 | 0.54 | - | - | - |  |
| VO_2_  (ml∙kg∙min^-1^) | 15.7±3.5 | 12.1±3.5 | 0.009 | 1.00 | 13.4±3.2 | 10.3±1.9 | 0.003 | 1.18 | 21.5±4.2 | 18.3±4.2 | 0.052 | 0.74 |
| Power output (watts) | 41.1±10.6 | 32.3±10.9 | 0.035 | 0.79 | 48±15.6 | 34.8±10.5 | 0.012 | 0.99 | 95.4±25.3 | 84.2±21.3 | 0.200 | 0.47 |
| HR  (bpm) | 156.7±15.1 | 143.7±25.9 | 0.118 | 0.57 | 124.2±10 | 109.2±14.7 | 0.004 | 1.13 | 167.2±22.9 | 160.1±22.9 | 0.407 | 0.30 |
| V_E_  (l∙min^-1^) | 28.9±6.6 | 26.3±9.5 | 0.402 | 0.30 | 21.9±6.5 | 21.1±5.3 | 0.710 | 0.13 | 45.9±18.5 | 45.6±11.1 | 0.958 | 0.01 |
| RER | 0.86±0.04 | 0.84±0.06 | 0.426 | 0.37 | 0.8±0.7 | 0.83±0.8 | 0.382 | 0.03 | 0.9±0.11 | 0.94±0.13 | 0.365 | 0.31 |
| % age-predicted HRmax | 83.2±10.5 | 76.3±11.9 | 0.109 | 0.59 | 75.6±12.4 | 68.8±9.2 | 0.098 | 0.61 | 88.7±13.8 | 85.1±9.6 | 0.411 | 0.30 |

Abbreviations: HRmax, maximum heart rate (age predicted = 220 minus age in years); RER, respiratory exchange ratio; V_E_, pulmonary ventilation; VO_2_, oxygen uptake; VO_2peak_, peak oxygen uptake. * Average values for all variables correspond to the highest 10-second value of HR obtained during the constant-load test for second wind detection (which on average was reached at 6 min 35 s ± 94 s).
